# A Comparison Between the Transoral Endoscopic Thyroidectomy Vestibular Approach and the Transareolar Approach Regarding Perioperative Complications: A Systematic Review and Meta-Analysis

**DOI:** 10.7759/cureus.56438

**Published:** 2024-03-19

**Authors:** Hyder Mirghani, Bandar Ahmed Alamrani, Mohammad Omar Aljabri, Fadi Olyan Alamrani, Meshal Saleh Alatawi, Meshari Mohammed Albalawi, Mohammed Abdullah S Alasmari, Ali Fahad B Alsharif, Waleed Muslih B Albalawi, Omar Sabbah Alzamhari

**Affiliations:** 1 Internal Medicine, University of Tabuk, Tabuk, SAU; 2 ENT Department, King Fahad Specialist Hospital, Ministry of Health, Tabuk, SAU; 3 General Medicine, King Khalid Hospital, Tabuk, SAU; 4 College of Medicine, University of Tabuk, Tabuk, SAU

**Keywords:** transoral endoscopic thyroidectomy, transareolar approaches, thyroidectomy complications, short-term complications, operative, vestibular approach

## Abstract

The global adoption of remote thyroidectomy is increasing, with the transoral endoscopic thyroidectomy vestibular approach (TOETVA) and transareolar approach (TAA) emerging as predominant methods. However, existing meta-analyses comparing these approaches to operative surgeries and short-term postoperative complications have significant limitations. To address this gap, our meta-analysis provides a comprehensive comparison between the TOETVA and TAA, focusing on operation time, intraoperative blood loss, postoperative drainage, and hospital stay duration. It aims to offer robust insights into their relative efficacy and safety profiles. We searched SCOPUS, PubMed, Web of Science, MEDLINE, and Cochrane Library from June 2015 to January 2024 for studies comparing transoral endoscopic thyroidectomy with the vestibular approach and areolar thyroidectomy using keywords, including "transoral thyroidectomy" and "scarless thyroidectomy." Studies were included if they were randomized controlled trials, case-control studies, or prospective/retrospective cohort studies comparing the TOETVA and TAA. Exclusion criteria removed case series, cross-sectional studies, editorials, non-English language, animal studies, and irrelevant articles. Data on operative time, postoperative drainage, intraoperative blood loss, and hospital stay were extracted. The Newcastle-Ottawa Scale was used to assess study quality (all studies scored 7-8). The findings revealed that the operative time was longer among the TOETVA group, with less intraoperative blood loss (odds ratio (OR) = 13.31, 95% confidence interval (CI) = 4.44-22.19); OR = -1.61, 95% CI = -2.82 to -0.39, respectively). Regarding hospitalization duration and postoperative drainage, no discernible difference was observed between the endoscopic TAA (ETAA) and TOETVA (OR = -0.04, 95% CI = -0.24 to 0.16; OR = -6.74, 95% CI = -20.08 to 6.60, respectively). The TOETVA has advantages over the TAA in terms of intraoperative blood loss and shorter operation times. However, both approaches exhibited comparable outcomes in terms of hospital stay duration and postoperative drainage. Furthermore, extensive randomized trials are warranted.

## Introduction and background

In recent years, there has been a global increase in the prevalence of thyroid nodules, with ultrasound detection accounting for 68% of cases in the general population [1]. Among these cases, well-differentiated thyroid cancer constitutes 5% to 10% [2]. Based on data from the Global Cancer Observatory (GLOBOCAN) 2020, thyroid cancer is ranked ninth among both sexes, with 586,202 newly diagnosed cases reported globally [3]. Follicular and papillary carcinoma, the two main histological types, comprise over 90% of well-differentiated thyroid cancers. Overall, thyroid cancer that is well-differentiated demonstrates a favorable prognosis, possessing a survival rate of 20 years [4].

Surgery remains the main treatment for patients with thyroid cancers and nodules [2]. In the past two decades, several remote access techniques have emerged for thyroidectomy [5]. Among them, the transoral endoscopic thyroidectomy vestibular approach (TOETVA) has gained extensive adoption globally due to its surgical effectiveness, cosmetic benefits, and low complication rate [6]. This distant technique is appropriate for treating both thyroid cancers and benign thyroid nodules [7].

He et al.'s report on thyroidectomy anticipated that transoral vestibulectomy offers a feasible option for patients concerned about cosmetic results [8]. Previous clinical experiences have showcased the remarkable efficacy of the TOETVA in managing hyperthyroidism and benign thyroid tumors, often leading to high levels of patient satisfaction [9]. The TOETVA is performed through two incisions on the lateral lower lip, and then the tissue is dissected through the anterior neck to reach the thyroid gland [10].

Likewise, the TOETVA and endoscopic transareolar approach (ETAA) have also emerged as successful techniques in the treatment of thyroid nodules or papillary carcinomas [11]. It is associated with minimal blood loss and shorter surgical times [11]. The transareolar technique approaches the thyroid gland through bilateral incisions in the areolar of the breast [11]. The literature comparing transoral vestibulectomy and transareolar thyroidectomy is scarce. Two studies compared endoscopic transoral video-assisted thyroidectomy (ETAA) [12,13]. According to these studies, patients undergoing endoscopic thyroidectomy via the oral vestibular approach (ETVOA) reported significantly higher cosmetic satisfaction due to the scarless approach [13]. In addition, the ETVOA resulted in less postoperative neck numbness, likely due to minimized nerve damage [13]. While ETVOA surgery times were longer than ETAA, potentially due to a learning curve for surgeons, the overall benefits for patient quality of life (QOL) likely outweighed this drawback [13]. Another research has also shown longer operative time, lesser intraoperative blood loss, and no significant postoperative drainage with ETVOA in comparison with ETAA [10].

While the literature comparing the TOETVA and ETAA is scarce, studies suggest higher cosmetic satisfaction and reduced postoperative complications with the TOETVA [12,13]. A meta-analysis comparing transareolar thyroidectomy and TOETVA could offer insights into operative characteristics, aiding in treatment decision-making. This meta-analysis aimed to compare transareolar thyroidectomy and TOETVA regarding operative characteristics (operative time, postoperative drainage, intraoperative blood loss, and hospital stay).

## Review

Materials and methods

Literature Search

A systematic literature search was carried out in SCOPUS, PubMed, Web of Science, MEDLINE, and Cochrane Library from July 2015 to January 2024. Fiver reviewers conducted a literature search to identify relevant articles using keywords: transareolar thyroidectomy, transoral thyroidectomy vestibular approach, scarless thyroidectomy, remote thyroidectomy, postoperative time, postoperative drainage postoperative blood volume, and hospital stay. In addition, the abstracts, references, and titles of the included studies were checked. After removing duplication, we found 391 papers and 278 stands. Of these, 65 full texts were examined, and nine studies were included in the final meta-analysis. These studies were published from 2015 to 2021.

Eligibility Criteria According to Population, Intervention, Comparison, Outcomes and Study (PICOS)

Inclusion and exclusion criteria: Studies were eligible if they were randomized controlled trials, case-control studies, and prospective and retrospective studies. The included studies must compare transareolar thyroidectomy and transoral thyroidectomy vestibular approach. Case series, cross-sectional studies, editorials, opinions, inadequate data, non-English language, animal studies, duplicate publications, and non-relevant studies were excluded.

Outcome Measures

The outcome measures were operative time, postoperative drainage, intraoperative blood loss, and hospital stay in days.

Data Extraction and Quality of Assessment

A datasheet was used to extract the data regarding study year, country of publication, author’s name, operative time, operative time, postoperative drainage, intraoperative blood volume, and hospital stay. The Newcastle-Ottawa Scale assessed the quality of the included studies, and all the studies were of good quality with a range of seven to eight [14].

Statistical Analysis

All statistical analyses were conducted using the most recent version of the RevMan software (Review Manager (RevMan), Version 5, The Cochrane Collaboration, 2020, available at revman.cochrane.org). For each outcome variable (operative time, intraoperative blood loss, postoperative drainage, and hospital stay), descriptive statistics were calculated to summarize the basic characteristics of the included studies. Mean values with standard deviations were reported for continuous variables, while percentages were provided for categorical variables.

The odds ratio (OR) with 95% confidence intervals (CIs) was calculated for dichotomous variables (operative time and postoperative drainage) using the Mantel-Haenszel method. For continuous variables (intraoperative blood loss and hospital stay), the weighted mean difference (WMD) with 95% CI was calculated using the inverse variance method.

Heterogeneity among the included studies was assessed using the I^2^ statistic and Cochran's Q test. Significant heterogeneity was considered present if the I^2^ value was greater than 50% or the Cochran's Q test had a p-value less than 0.05. The fixed-effects model was applied when heterogeneity was not significant, while the random-effects model was used in the presence of significant heterogeneity. The choice between the fixed-effects and random-effects models was based on the assumption that the effect sizes of the included studies were either identical (fixed-effects) or varied (random-effects). Subgroup analyses or sensitivity analyses were performed if substantial heterogeneity was detected to discover potential sources of heterogeneity. In addition, publication bias was assessed using funnel plots and Egger's regression test. A p-value less than 0.05 was considered statistically significant for all analyses.

Results

**Figure 1:**
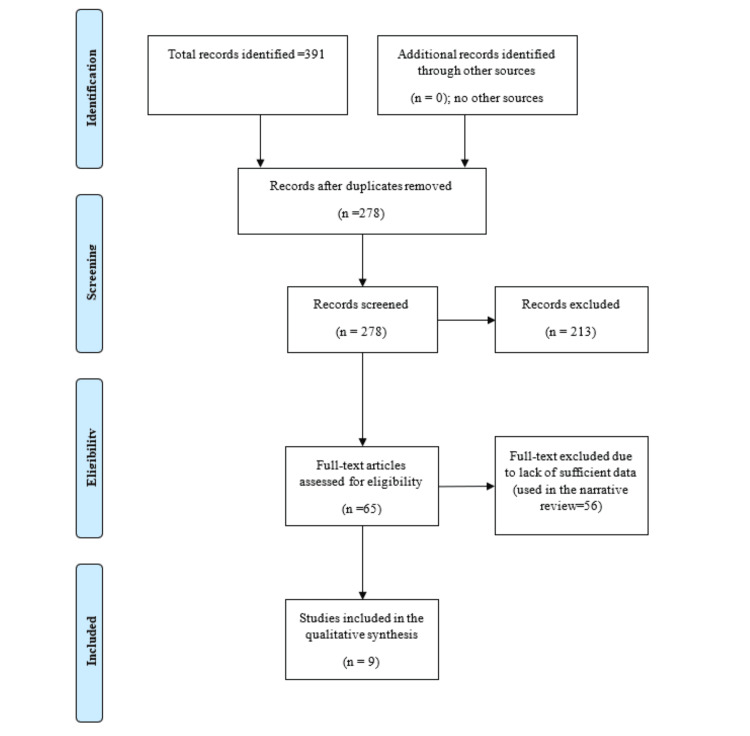
PRISMA flowchart for the comparison of transareolar thyroidectomy and transoral thyroidectomy vestibular approach regarding operative and short-term postoperative complications

The literature search is shown in Figure 1. Of the 391 studies that were found, 278 remained after duplication was removed. Of these, 65 full texts were evaluated, and nine papers were included in the final meta-analysis.

Table 1 illustrates a comprehensive overview of the basic patient characteristics across the included studies. Predominantly conducted in Asia, with one study from Europe, the age of the participants ranged from 29.8 to 41.2 years. Female patients were predominant in most studies, with lobectomy being the most common surgical procedure, primarily for papillary microcarcinoma. This table facilitates understanding the demographic distribution and surgical indications among the populations studied.

**Table 1:** Basic characteristics of patients with transoral approach and areolar thyroidectomy.

Author	Country	Age/years (mean ± SD)	Females (frequencies)	Type of operation	Pathology
Ding et al., 2017 [15]	China	33.1 ±2.4 vs. 34.2 ±2.6	50% vs. 60%	Thyroidectomy	Thyroid disease
Guo et al., 2019 [16]	China	29.8±0.96 vs. 33.75±1.19	All women	Lobectomy	Papillary thyroid microcarcinomas
Hou et al., 2019 [17]	China	32.54±7.59 vs. 33.83±8.10	78.9% vs. 60%	Lobectomy	Unilateral papillary thyroid carcinoma.
Shen et al., 2021 [18]	China	37.8 ± 12.4 vs. 41.2 ± 11.9	63.2% vs. 64.9%	Lobectomy	Benign thyroid nodules
Sun et al., 2016 [19]	China	29.65 ± 6.57 vs. 34.59 ± 7.69	86% vs. 86.5%	Lobectomy	Papillary thyroid carcinomas <2cm
Xu et al., 2019 [20]	China	30.46 ±6.93 vs. 33.3 ±6.94	91.7% vs. 88.6%	Lobectomy	Papillary thyroid microcarcinomas
Yang et al., 2015 [21]	China	31.9±8.8 vs. 31.0±8.9	75.8% vs. 63.3%	Thyroidectomy	Thyroid disease
Zhang et al., 2020 [22]	China	22.2±3.0 vs. 23.7±3.8	91.7% vs. 100%	Lobectomy	Unilateral papillary thyroid carcinoma
Zhang et al., 2021 [23]	China	33.4±6.8 vs. 34.4±7.6	70.4% vs. 100%	Lobectomy	Unilateral papillary thyroid carcinoma.

**Figure 2:**
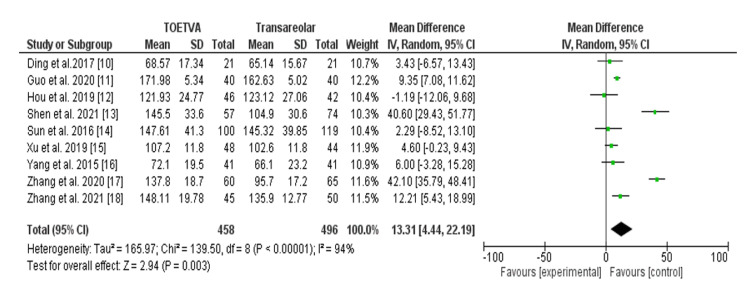
Operative time (minutes) among patients with transoral approach and areolar thyroidectomy. TOETVA: transoral endoscopic thyroidectomy vestibular approach, SD: standard deviation

Table 2 compares operative times in minutes between the transoral vestibular approach and areolar thyroidectomy across nine studies [15-23], encompassing 954 patients. Notably, the TOETVA generally exhibited longer operative times compared to the areolar approach, with an odds ratio (OR) of 13.31 (95% confidence interval (CI), 4.44-22.19). However, significant heterogeneity (I^2^ = 94%, p < 0.001) suggests substantial variability in study results, as shown in Figure 2.

**Table 2:** Operative time (minutes) among patients with the transoral approach and areolar thyroidectomy TOETVA: transoral endoscopic thyroidectomy vestibular approach, SD: standard deviation

Author	Country	Study type	TOETVA (Mean ± SD)	Areolar (Mean ± SD)
Ding et al., 2017 [15]	China	Prospective	68.57 ± 17.34	65.14 ± 15.67
Guo et al., 2019 [16]	China	Retrospective	171.98 ± 5.34	162.63 ± 5.02
Hou et al., 2019 [17]	China	Retrospective	121.93 ± 24.77	123.12 ± 27.06
Shen et al., 2021 [18]	China	Prospective	145.5 ± 33.6	104.9 ± 30.6
Sun et al., 2016 [19]	China	Retrospective	147.61 ± 41.30	154.32 ± 39.85
Xu et al., 2019 [20]	China	Retrospective	107.2 ± 11.8	102.6 ± 11.8
Yang et al., 2015 [21]	China	Retrospective	72.1 ± 19.5	66.1 ± 23.2
Zhang et al., 2020 [22]	China	Retrospective	137.8 ± 18.7	95.7 ± 17.2
Zhang et al., 2021 [23]	China	Retrospective	148.11 ± 19.78	135.90 ± 12.77

**Figure 3:**
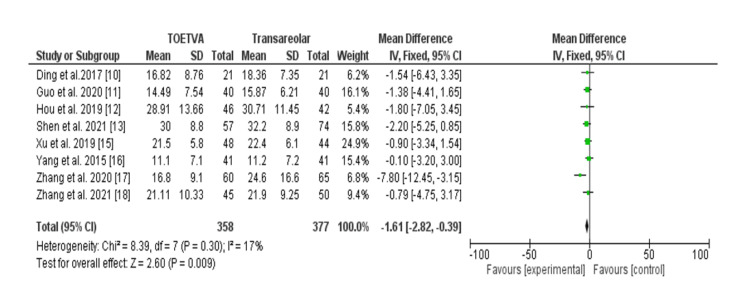
Intraoperative blood loss (ml) among patients with transoral approach and areolar thyroidectomy. TOETVA: transoral endoscopic thyroidectomy vestibular approach, SD: standard deviation

Table 3 outlines intraoperative blood loss (ml) among patients undergoing the transoral vestibular approach (TOETVA) versus areolar thyroidectomy across eight studies, encompassing 735 patients. Notably, patients undergoing the TAA experienced lower intraoperative blood loss compared to those undergoing the transoral vestibular approach, with an OR of -1.61 (95% CI: -2.82 to -0.39). Statistical analysis revealed non-significant heterogeneity (I^2^ = 17%, p = 0.30), indicating consistent results across studies. Figure 3 shows the forest plot presentation.

**Table 3:** Intraoperative blood loss (ml) among patients with the transoral approach and areolar thyroidectomy TOETVA: transoral endoscopic thyroidectomy vestibular approach, SD: standard deviation

Author	Country	Study type	TOETVA (Mean ± SD)	Areolar (Mean ± SD)
Ding et al., 2017 [15]	China	Prospective	16.82 ± 8.76	18.36 ± 7.35
Guo et al., 2019 [16]	China	Retrospective	14.49 ± 7.54	15.87 ± 6.21
Hou et al., 2019 [17]	China	Retrospective	28.91 ± 13.66	30.71 ± 11.45
Sun et al., 2021 [18]	China	Prospective	30.0 ± 8.8	32.2 ± 8.9
Xu et al., 2019 [20]	China	Retrospective	21.5 ± 5.8	22.4 ± 6.1
Yang et al., 2015 [21]	China	Retrospective	11.1 ± 7.1	11.2 ± 7.2
Zhang et al., 2020 [22]	China	Retrospective	16.8 ± 9.1	24.6 ± 16.6
Zhang et al., 2021 [23]	China	Retrospective	21.11 ± 10.33	21.90 ± 9.25

**Figure 4:**
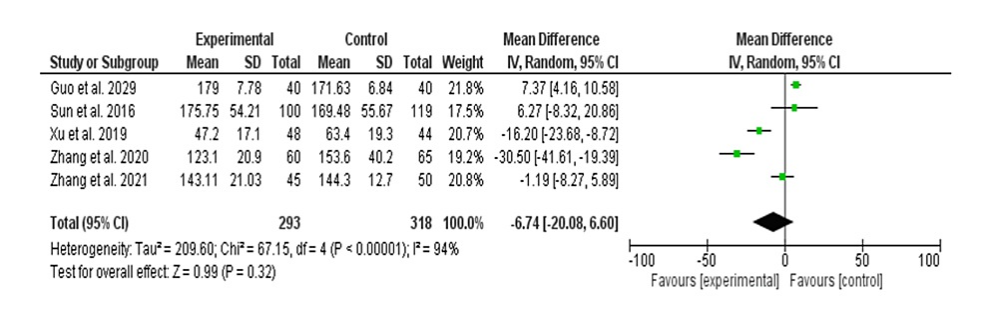
Postoperative drainage (ml) among patients with the transoral approach and areolar thyroidectomy. TOETVA: transoral endoscopic thyroidectomy vestibular approach, SD: standard deviation

Table 4 shows the postoperative drainage (ml) in the transoral approach and areolar thyroidectomy. The postoperative drainage volume was not significantly different between the two approaches, with OR= -6.74, 95% CI= (-20.08-6.60). The chi-square was 67.15, significance tests for the weighted average effect size (Z) = 0.99, and the p-value for the overall effect = 0.32. Significant heterogeneity was identified, with an I^2^ value of 94%, a p-value below 0.001, and a standard deviation of 4. Figure 4 shows the forest plot presentation.

**Table 4:** Postoperative drainage (ml) among patients with the transoral approach and areolar thyroidectomy TOETVA: transoral endoscopic thyroidectomy vestibular approach, SD: standard deviation

Author	Country	Study type	TOETVA (Mean ± SD)	Areolar (Mean ± SD)
Guo et al., 2029 [12]	China	Retrospective	179.00 ± 7.78	171.63 ± 6.84
Shen et al., 2016 [16]	China	Retrospective	175.75 ± 54.21	169.48 ± 55.67
Xu et al., 2019 [20]	China	Retrospective	47.2 ± 17.1	63.4 ± 19.3
Zhang et al., 2020 [22]	China	Retrospective	123.1 ± 20.9	153.6 ± 40.2
Zhang et al., 2021 [23]	China	Retrospective	143.11 ± 21.03	144.3 ± 12.70

**Figure 5:**
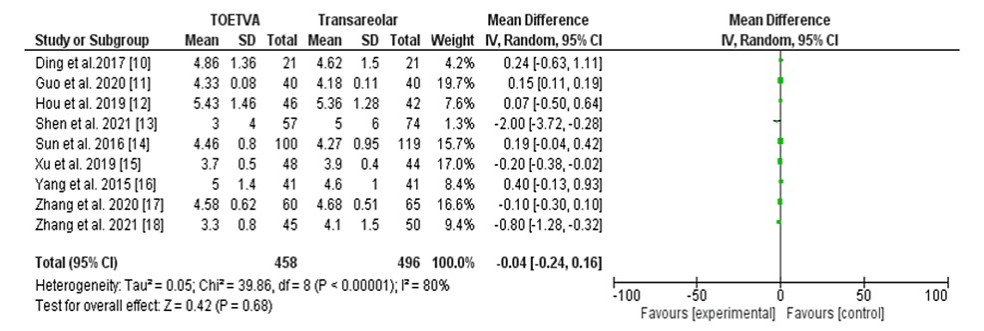
Hospital stays (days) among the patients with the transoral approach and areolar thyroidectomy. TOETVA: transoral endoscopic thyroidectomy vestibular approach, SD: standard deviation

Table 5 illustrates the duration of hospital stay (days) comparing the transoral vestibular approach and areolar thyroidectomy across nine studies [15-23], comprising 954 patients. No significant difference was observed in hospital stay duration, with an OR of -0.04 (95% CI: -0.24 to 0.16). However, significant heterogeneity was noted (I^2^ = 80%, p < 0.001), suggesting variability among study findings. Figure 5 shows the forest plot presentation.

**Table 5:** Hospital stay (days) among patients with the transoral approach and areolar thyroidectomy TOETVA: transoral endoscopic thyroidectomy vestibular approach, SD: standard deviation

Author	Country	Study type	TOETVA (Mean ± SD)	Areolar (Mean ± SD)
Ding et al., 2017 [15]	China	Prospective	4.86 ± 1.36	4.62 ± 1.5
Guo et al., 2029 [16]	China	Retrospective	4.33 ± 0.08	4.18 ± 0.11
Hou et al., 2019 [17]	China	Retrospective	5.43 ± 1.46	5.36 ± 1.28
Shen et al., 2021 [18]	China	Prospective	3 ± 4	5 ± 6
Sun et al., 2016 [19]	China	Retrospective	4.46 ± 0.80	4.27 ± 0.95
Xu et al., 2019 [20]	China	Retrospective	3.7 ± 0.5	3.9 ± 0.4
Yang et al., 2015 [21]	China	Retrospective	5.0 ± 1.4	4.6 ± 1.0
Zhang et al., 2020 [22]	China	Retrospective	4.58 ± 0.62	4.68 ± 0.51
Zhang et al., 2021 [23]	China	Retrospective	3.3 ± 0.8	4.1 ± 1.5

Discussion

The present meta-analysis encompassed nine studies involving 954 patients to compare the outcomes of the transoral vestibular approach (TOETVA) versus the areolar approach (ETAA) in endoscopic thyroid surgery. One of the notable findings is the longer operative time associated with the TOETVA compared to the ETAA, consistent with previous studies [24]. Similarly, a study by Wang et al. compared the TOETVA and conventional thyroidectomy and found a long operative time in the TOETVA [10]. A more recent meta-analysis also confirmed the long operating time in the TOETVA compared to non-transoral endoscopic approaches [25]. This disparity in operative time between the TOETVA and ETAA may be attributed to several factors, including the narrower operating space inherent to the transoral vestibular approach and the surgeon's proficiency level, as suggested in prior research. It is well-documented that enhanced surgical skill and experience can lead to shorter operation times, as demonstrated in prior studies [26,27]. In addition, intraoperative blood loss was lower in patients undergoing the ETAA, as indicated by eight studies involving 735 patients. The current findings were similar to Xia et al. [10]. In their study, intraoperative blood loss was found to be lower in ETAA compared to conventional thyroidectomy. However, Xia et al. were greatly limited by including two studies that compared substernal goiter with trainsareolar and trans-subclavian approaches rather than the transoral [28,29]. Overall, this discrepancy in blood loss could be influenced by anatomical differences and surgical techniques between the two approaches.

Interestingly, according to our nine studies, postoperative drainage volume did not significantly differ between the two approaches, despite the absence of drainage tubes in the TOETVA group. This finding is supported by research conducted by de Vries et al. in which no difference was noted in drainage volume [30]. However, de Vries and colleagues compared endoscopic and minimally invasive thyroidectomy either with each other or with conventional thyroidectomy.

Furthermore, no notable differences were observed in hospital stay between the TOETVA and ETAA groups, underscoring the comparable postoperative recovery trajectories in terms of length of hospitalization. The limitations of the study included the small sample size of the included studies. In addition, the studies were observational and were conducted solely in a single Asian country. However, a great uptake was observed in the Western world. The European Transoral Endoscopic Thyroidectomy Vestibular Approach (ETOETVA) Study Group has just published a study from nine centers in four European countries [30].

Overall, this meta-analysis suggests that the ETAA offers less intraoperative blood loss and shorter operative times compared to the TOETVA. There seems to be no significant difference between the two approaches in terms of postoperative drainage and hospital stay. However, the high heterogeneity across studies highlights the need for more high-quality research with longer follow-up periods and ideally randomized controlled trials. This would provide a more definitive comparison of these scarless thyroidectomy techniques.

The findings from this meta-analysis have several implications for clinical practice. First, clinicians should consider the benefits of the ETAA over the TOETVA, particularly in terms of reduced intraoperative blood loss and shorter operative times. However, it is crucial to note that no significant differences were observed in postoperative drainage and hospital stay between the two approaches, suggesting comparable outcomes in terms of postoperative recovery trajectories.

Nevertheless, the limitations of the study, such as the small sample size and the observational nature of the included studies, must be acknowledged. In addition, the studies were predominantly conducted in a single Asian country, although recent uptake in the Western world, as evidenced by the ETOETVA Study Group's publication, highlights a growing interest in these techniques globally.

Clinicians should interpret these findings cautiously, recognizing the high heterogeneity across studies and the need for more high-quality research with longer follow-up periods, ideally in the form of randomized controlled trials. This would provide a more definitive comparison of these scarless thyroidectomy techniques and inform clinical decision-making regarding the optimal approach for patients undergoing thyroid surgery.

## Conclusions

The endoscopic thyroidectomy areolar approach was better compared to the TAA regarding intraoperative blood loss and shorter operation times. No differences were evident regarding postoperative drainage and hospital stay. Further longer randomized trials are needed for a preoperative shared decision.

## References

[1] Kant R, Davis A, Verma V (2020). Thyroid nodules: advances in evaluation and management. Am Fam Physician.

[2] Iyer NG, Shaha AR (2010). Management of thyroid nodules and surgery for differentiated thyroid cancer. Clin Oncol (R Coll Radiol).

[3] Sung H, Ferlay J, Siegel RL, Laversanne M, Soerjomataram I, Jemal A, Bray F (2021). Global Cancer Statistics 2020: GLOBOCAN estimates of incidence and mortality worldwide for 36 cancers in 185 countries. CA Cancer J Clin.

[4] Ajani JA, D'Amico TA, Almhanna K (2016). Gastric cancer, Version 3.2016, NCCN clinical practice guidelines in oncology. J Natl Compr Canc Netw.

[5] Anuwong A (2016). Transoral endoscopic thyroidectomy vestibular approach: a series of the first 60 human cases. World J Surg.

[6] Kim SY, Kim SM, Makay Ö (2020). Transoral endoscopic thyroidectomy using the vestibular approach with an endoscopic retractor in thyroid cancer: experience with the first 132 patients. Surg Endosc.

[7] Anuwong A, Ketwong K, Jitpratoom P, Sasanakietkul T, Duh QY (2018). Safety and outcomes of the transoral endoscopic thyroidectomy vestibular approach. JAMA Surg.

[8] He Q, Zhu J, Li X (2022). A comparative study of two robotic thyroidectomy procedures: transoral vestibular versus bilateral axillary-breast approach. BMC Surg.

[9] Yang C, Yu L, Xiao Y, Ouyang L, Huang X (2022). Discussion on the transoral vestibular approach endoscopy for TC patients and the significance of serum 25-hydroxyvitamin D classification. J Healthc Eng.

[10] Xia B, Xing Z, Qiu Y (2023). Endoscopic thyroidectomy via oral vestibular approach versus areolar approach: a meta-analysis. Asian J Surg.

[11] Shen S, Hu X, Qu R, Guo Y, Luo L, Chen X (2021). Comparing quality of life between patients undergoing trans-areola endoscopic thyroid surgery and trans-oral endoscopic thyroid surgery. BMC Surg.

[12] Chai YJ, Chung JK, Anuwong A (2017). Transoral endoscopic thyroidectomy for papillary thyroid microcarcinoma: initial experience of a single surgeon. Ann Surg Treat Res.

[13] Wu GY, Fu JB, Lin FS, Luo YZ, Lin ED, Yan W (2017). Endoscopic central lymph node dissection via breast combined with oral approach for papillary thyroid carcinoma: a preliminary study. World J Surg.

[14] Stang A (2010). Critical evaluation of the Newcastle-Ottawa scale for the assessment of the quality of nonrandomized studies in meta-analyses. Eur J Epidemiol.

[15] Rare cases for surgical branches [Article in Turkish] (2019). Akademisyen Kitabevi.

[16] Zhihong L, Yihong T, Weixin L (2021). A comparative study on the efficacy and safety of transoral vestibular approach and transthoracic breast approach for laparoscopic thyroid micropapillary carcinoma surgery. Chin J Anat Clin.

[17] Guo F, Wang W, Zhu X, Xiang C, Wang P, Wang Y (2020). Comparative study between endoscopic thyroid surgery via the oral vestibular approach and the areola Approach. J Laparoendosc Adv Surg Tech A.

[18] Sun H, Zheng H, Wang X, Zeng Q, Wang P, Wang Y (2020). Comparison of transoral endoscopic thyroidectomy vestibular approach, total endoscopic thyroidectomy via areola approach, and conventional open thyroidectomy: a retrospective analysis of safety, trauma, and feasibility of central neck dissection in the treatment of papillary thyroid carcinoma. Surg Endosc.

[19] Xu Z, Song J, Wang Y, Tan L, Sun S, Meng Y (2019). A comparison of transoral vestibular and bilateral areolar endoscopic thyroidectomy approaches for unilateral papillary thyroid microcarcinomas. Wideochir Inne Tech Maloinwazyjne.

[20] Yang J, Wang C, Li J (2015). Complete endoscopic thyroidectomy via oral vestibular approach versus areola approach for treatment of thyroid diseases. J Laparoendosc Adv Surg Tech A.

[21] Zhang WD, Dai L, Wang YC, Xie YY, Guo JY, Li JJ, Wu XJ (2021). Transoral endoscopic thyroidectomy vestibular approach versus endoscopic thyroidectomy via areola approach for patients with unilateral papillary thyroid carcinoma: a retrospective study. Surg Laparosc Endosc Percutan Tech.

[22] Zhang G, Li B, Zhang G, Lin Y, Chen Y, Gao J (2020). Pros and cons of transoral endoscopic thyroidectomy via vestibular approach: a comparative study. Surg Laparosc Endosc Percutan Tech.

[23] Shen S, Hu X, Qu R, Duo Y (2020). Trans-oral endoscopic thyroid surgery and trans-areola endoscopic thyroid surgery: which offers a better quality of life. BMC Surf.

[24] Wang D, Wang Y, Zhou S, Liu X, Wei T, Zhu J, Li Z (2022). Transoral thyroidectomy vestibular approach versus non-transoral endoscopic thyroidectomy: a comprehensive systematic review and meta-analysis. Surg Endosc.

[25] Razavi CR, Vasiliou E, Tufano RP, Russell JO (2018). Learning curve for transoral endoscopic thyroid lobectomy. Otolaryngol Head Neck Surg.

[26] Park JH, Lee J, Hakim NA (2015). Robotic thyroidectomy learning curve for beginning surgeons with little or no experience of endoscopic surgery. Head Neck.

[27] Liu Y, Li X, Liu H, Gao W, Zhao Y (200644). Endoscopic thyroid surgery: a comparison of the trans-subclavian and the trans-areolar approach. Chin J Surg.

[28] Wang C, Sun P, Li J (2016). Strategies of laparoscopic thyroidectomy for treatment of substernal goiter via areola approach. Surg Endosc.

[29] de Vries LH, Aykan D, Lodewijk L, Damen JA, Borel Rinkes IH, Vriens MR (2021). Outcomes of minimally invasive thyroid surgery - a systematic review and meta-analysis. Front Endocrinol (Lausanne).

[30] Woods RS, Woods JF, Duignan ES, Timon C (2014). Systematic review and meta-analysis of wound drains after thyroid surgery. Br J Surg.

